# Microalgae Bioactive Carbohydrates as a Novel Sustainable and Eco-Friendly Source of Prebiotics: Emerging Health Functionality and Recent Technologies for Extraction and Detection

**DOI:** 10.3389/fnut.2022.806692

**Published:** 2022-03-15

**Authors:** Mostafa Gouda, Musa A. Tadda, Yinglei Zhao, F. Farmanullah, Bingquan Chu, Xiaoli Li, Yong He

**Affiliations:** ^1^College of Biosystems Engineering and Food Science, Zhejiang University, Hangzhou, China; ^2^Department of Nutrition and Food Science, National Research Centre, Giza, Egypt; ^3^Department of Agricultural and Environmental Engineering, Faculty of Engineering, Bayero University, Kano, Nigeria; ^4^Institute of Agricultural Equipment, Zhejiang Academy of Agricultural Sciences, Hangzhou, China; ^5^Faculty of Veterinary and Animal Sciences, National Center for Livestock Breeding Genetics and Genomics LUAWMS, Uthal, Pakistan; ^6^School of Biological and Chemical Engineering, Zhejiang University of Science and Technology, Hangzhou, China

**Keywords:** microalgae, prebiotics, phytochemistry, exopolysaccharides, oligosaccharides, biotechnology, Spirulina and Chlorella products

## Abstract

There is a global interest in the novel consumption, nutritional trends, and the market of new prebiotic sources and their potential functional impacts. Commercially available nutritional supplements based on microalgae that are approved to be edible by FDA, like *Arthrospira platensis* (Cyanobacteria) and *Chlorella vulgaris* (Chlorophyta) become widely attractive. Microalgae are rich in carbohydrates, proteins, and polyunsaturated fatty acids that have high bioactivity. Recently, scientists are studying the microalgae polysaccharides (PS) or their derivatives (as dietary fibers) for their potential action as a novel prebiotic source for functional foods. Besides, the microalgae prebiotic polysaccharides are used for medication due to their antioxidant, anticancer, and antihypertensive bioactivities. This review provides an overview of microalgae prebiotics and other macromolecules’ health benefits. The phytochemistry of various species as alternative future sources of novel polysaccharides were mentioned. The application as well as the production constraints and multidisciplinary approaches for evaluating microalgae phytochemistry were discussed. Additionally, the association between this potential of combining techniques like spectroscopic, chromatographic, and electrochemical analyses for microalgae sensation and analysis novelty compared to the chemical methods was emphasized.

## Introduction

The international community’s attention has lately risen in a major way to the emerging market of dietary fibers and other phytochemical supplements, which are considered to be safe and have significant functional impacts. As an important example, microalgae are considered as future superfoods, as mentioned by Torres-Tiji et al. (1). They have several important nutritional phytochemicals such as phycocyanin (a novel protein in Spirulina), exopolysaccharides, polyphenols, and flavonoids.

In addition, many studies have reported that microalgae have high antiradical activity coming from their unique chemical structure (2). Furthermore, scientists are trying to find instant and non-invasive techniques to explore the scientific principles of microalgae phytochemicals’ functional principles, especially bioactive macromolecules and micromolcules (3). These phytochemicals increase the media antioxidant, anticancer, and other physicochemical potentials (4–9).

The microalgae health-related key components like carbohydrates (especially prebiotics), lipids, and proteins are precursors of essential pathways and are novel sources of bioactive molecules. As an example, microalgae polysaccharides, provide many intriguing aspects in the food science and nutrition area as a viable alternative in food technology. They are widely used in biopolymer-based materials, like alginate polysaccharides that is among the most versatile biodegradable polymers by microorganisms (10). Also, *Phaeodactylum tricornutum* produces up to 40% eicosapentaenoic acid (EPA) and docosahexaenoic acid (DHA) of its fatty acids, as a functional bioactive molecule (1). Additionally, microalgae are recently used as a novel proteins source. These proteins include commercially available phycocyanin novel food protein supplements produced by Spirulina (*A. platensis*; or Cyanobacteria) (11).

As their potential uses as functional food, microalgae prebiotics have novel characteristics distinguished from the other edible sources against the digestion by the gastrointestinal tract. Therefore, they help to enhance the growth of the health-related beneficial bacterial microorganisms called probiotics in the lower part of the gastrointestinal tract or the colon (12). Meanwhile, it could be used as a symbiotic (probiotics and prebiotics) functional food, for improving their positive health effects. In which, there are a large number of probiotic bacteria in dairy products that proved their efficiency when used in a mixture with the prebiotic fibers for improving the gut health in the different cultures traditional foods, like the fermented Sobya and Kefir (5, 6). Also, this recently explored prebiotics from microalgae confirmed their potentials to support probiotic beneficial bacterial growth in the host gut upon consumption (13). The most promising polysaccharides (PS) from microalgae (like exopolysaccharides, alginates, and carrageenans) are barely fermented by the colonic microbiota, therefore they act as efficient prebiotics. Moreover, the microalgae prebiotic functionality are significantly affected by industrial processes impacts on its chemical composition (14). Currently, chemical and chromatographic methods are used for the evaluation and analysis of microalgae chemical composition. However, these methods are destructive and are chemical dependencies that affect their structure (15). On the other hand, some potential novel examples for detecting single-cell microalgae macromolecules functionality could be through its electrochemical charge that is a very important parameter for identifying and establishing a reference method for its characterization based on electronegativity fingerprint (16–18).

The aims of this review was to present and highlight the potential uses of microalgae, as alternative prebiotic edible sources for human consumption. Furthermore, the advanced non-destructive techniques were summarized for facilitating the tracking of these functional molecules’ during its cultivation process.

## Microalgae as a Sustainable and Eco-Friendly Source

Microalgae species have the potential to be a sustainable high nutritious sources. In which, several microalgae species are generally recognized as safe (GRAS) (1), like *Arthrospira platensis*, and *Scenedesmus obliquus*. They are well known in United States, China, and Japan market (2, 19). Microalgae are unicellular cells that are considered eukaryotic, non-flowering, and photosynthetic aquatic plants sometimes. These microorganisms can act independently or with some other formulations to improve human health (2, 20).

Several large-scale aquaponics and Hydroponics plant systems have developed and used microalgal technologies to reuse the organic and inorganic molecules in their aquatic culture (21). Microalgae biotechnology have several advantages compared to the other microorganisms. For instance, they could change their metabolic pathways in the production of new novel functional compounds which open the way to use these kind of safe source instead of other known sources like bacteria for the production of novel molecules (22). Furthermore, its high biosorption ability of the essential elements like Mn (II) (e.g., *Thalassiosira pseudonana*) through its active hybrid membrane system performance let it be used in several advanced pharmaceutical field for optimizing their production of prebiotics like exopolysaccharides (EPS) (23), as anticancer, anti-inflammatory, and immunomodulatory agent (24).

## Microalgae Healthy Macromolecules and Phytochemicals

**FIGURE 1:**
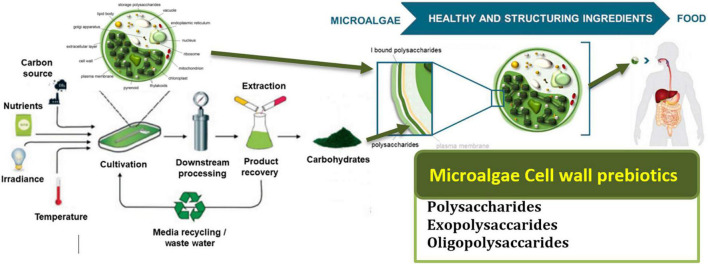
Presents the cycle of microalgae from their production to consumption in the human body and their potential benefits. Adapted from Agarwal, Gupta, Biswas and Systems (121) (Copyright permission: 5179150775488). Created with Adobe illustrator.

Table 1 presents the edible marine microalgae carbohydrate, oil, and protein contents, in which it can be observed that *Scenedesmus sp.* contains up to 41%, *Porphyridium* contains up to 57%, and *Chlorella* contains up to 26% of carbohydrates that contains many potential prebiotic bioactive. On the other hand, *Spirulina Maxima* contains up to 71% protein and *Phaeodactylum sp.* contains up to 57% of oil. Thus, microalgae, in general, could be used as perfect sustainable alternative food that have health benefits for humans (25). And therefore, Qiao et al. (26) reported that these naturally existing and very-fast growing unicellular microorganisms’ phytochemicals have been applied in the medical area to increase oxygen local level in tumor regions (Figure 1).

**TABLE 1:** Oil, protein, and carbohydrate contents of marine microalgae expressed on a dry biomass.

Microalgae	Lipid and oil (%)	Protein (%)	Carbohydrate (%) include prebiotics	Total phenolics (mg/g)	Total flavonoids (mg/g)	Antioxidant activity (μg/g) Gallic equ.	References
*Phaeodactylum tricornutum*	18–57	30	8.4	10.46	15.29	54.02	(2, 25)
*Dunaliella. salina*	6–25	57	32	–	–	–	(25)
*Scenedesmus obliquus*	30–50	10–45	20–40	3.55	4.42	6.58	(2, 25)
*Chlorella vulgaris*	28–53	25–45	24–30	5.87	5.47	15.10	(2, 25)
*Nostoc commune*	22	20.3–43	34–56.4	–	–	–	(25)
*Spirulina Platensis*	4–11	46–63	8–14	7.59	9.26	26.02	(2, 25)
*Porphyridium cruentum*	9–14	28–39	40–57	–	–	–	(25)
*Schizochytrium limacinum*	43	39	5	–	–	–	(25)

As an example, microalgae have high concentrations of the polyunsaturated fatty acids (PUFAs), which are category of fatty acids containing more than one double bond in the backbone and which have high bioactivity against cholesterol levels in the human body (27, 28) (Table 2).

**TABLE 2:** Screening of algal strains with the potential for PUFAs production (27, 28).

Microalgae species	Algae growth condition	Biomass yield or productivity (mg/L/day)	Lipid content (%)	EPA (%)	DHA (%)	PUFA (%)
*Arcocellulus cornucervis*	Marine and Cold environment	8.67	–	1.36	0.23	–
*Phaeodactylum tricornutum*		60.00	–	3.14	0.25	–
*Attheya septentrionalis*		22.22	–	4.58	0.60	–
*Thalassiosira hispida*		12.86	–	4.10	0.47	–
*Scenedesmus dimorphus*		14.0	–	–	–	39.03
*Monodus subterraneus*		450–1420	–	–	–	–
*Parietochloris incisa*		–	–	–	–	–
*Attheya septentrionalis*		0.54–0.57	–	7.1	–	–
*Botryococcus braunii*	Trophic regions with low latitude	158.9	41.98	–	–	11.49
*Chlorella sp.*		249.2	21.54	–	–	55.52
*Chlorococcum humicola*		198.3	29.16	–	–	14.07
*Chlamydomonas sp.*		236.8	21.92	–	–	36.27
*Fistulifera sp.*	Marine diatom	–	–	17.1	–	–
*Synedra fragilaroides*	Marine diatom	430	0.43	11.0	2.1	36.80
*Nitzschia closterium*		100	0.10	19.5	1.1	33.00
*Phaeodactylum tricomutum*		220	0.22	8.8	0.5	14.90

*Individual fatty acids are a percentage of EPA: Eicosapentaenoic acid; DHA, docosahexaenoic acid; PUFA, polyunsaturated fatty acid; “–” refers to “not available”.*

Also, microalgae can scavenge a wild range of Reactive Oxygen Species (ROS) through their high phenolic and flavonoid content. For instance, our recent study on four microalgae species confirmed their high potential as antioxidant agents (Table 1). An innovative way of *in situ* oxygen-generation by an engineered *C. vulgaris* to overcome hypoxia in wounds was reported by Qiao et al. (26), whereby the engineered live *C. vulgaris* added to hypoxic tumor areas has both increased local oxygen levels and re-sensitized resistant cancer cells via microalgae-mediated photosynthesis under red-light beams. This engineered- *C. vulgaris* was achieved by adding an engineered red blood cell membrane (RBCM) to the *C. vulgaris* surface which reduces macrophage uptake and systemic clearance of the *C. vulgaris* and was termed as “RBCM-Algae.” The significant effect of *C. vulgaris* was reported through its *in situ* generation of O_2_ in the human’s system using a natural photosynthation. In which, the increased oxygen level on the tumor areas by the RBCM-Algae resulted in a remarkably radiotherapeutic efficacy. Besides, the chlorophyll from the *C. vulgaris* was observed to produce ROS during laser irradiation adding to improved tumor cells’ apoptosis (26).

**FIGURE 2:**
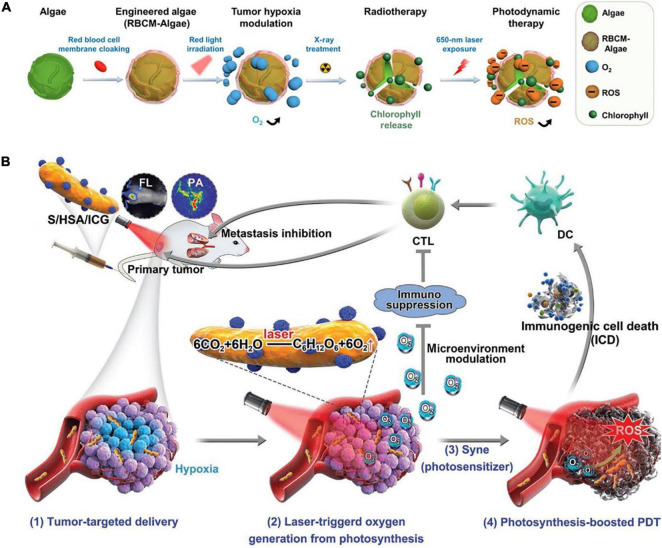
**(A)** Demonstration of the microalgae engineered treatments. **(B)** Human serum albumin (HSA)/indocyanine green (ICG) *in situ* generated O_2_ through photosynthesis to enhance photodynamic therapy (PDT) efficacy and induce immunogenic cell death (ICD)-mediated antitumor immune activities (122) (Copyright permission: 5179180996104).

Furthermore, it was demonstrated that the engineered- *C. vulgaris* when injected into the body by either intravenous or intratumoral, could efficiently arrive at the tumor spot and improve oxyhemoglobin, as it shows an extended life span with increased stability during *in vivo* circulation, which exhibits excellent biocompatibility with tissue cells (26). Furthermore, when compared to most normal tissues that were equally inoculated with the RBCM-Algae, such as the brain, heart, and kidney tissues, the tumor tissues showed the highest uptake of the RBCM-Algae (Figure 2). Therefore, the microalgae functional components could give it a high selectivity when they are used as a prebiotic functional source in the human body (26).

**FIGURE 3:**
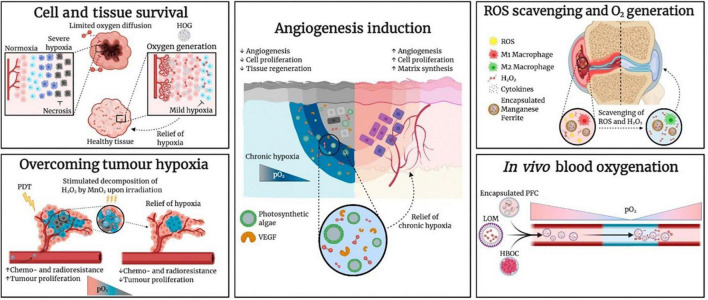
Research Applications of Oxygen-Carrying and Oxygen-Generating Biomaterials (OCBs and OGBs) include the microalgae. Hemoglobin-based oxygen carrier (HBOC), hydrophobic oxygen generator (HOG), lipid-based oxygen microbubble (LOM), photodynamic therapy (PDT), perfluorocarbon (PFC), partial pressure of oxygen (pO_2_), and vascular endothelial growth factor (VEGF) (31) (Copyright permission: 5179181247874).

In another recent practical study of the health-related impacts of microalgae and their functional components by Chen et al. (29), an oxygen-producing-patch filled with gel beads containing active *Synechococcus elongatus* was reported to have efficiently supplied more than 100 times sufficient oxygen than the available conventional ways of supplying oxygen, such as hyperbaric oxygen (HBO), topical dissolved oxygen (TDO), and topical gaseous oxygen (TGO) (Figure 3). For example, based on the level of oxygen penetration to the dermis, TDO and TGO had recorded > 700 and only 300 μm of human skin, respectively, demonstrating TDO as the best available method to supply oxygen to the chronic diabetic wounds. In which, its mechanism is based on producing continuous oxygen by the photosynthesis process, which can effectively penetrate the skin to stimulate aerobic metabolism and angiogenesis in hypoxic tissues (30). Based on these excellent qualities demonstrated by *S. elongatus* gel-wound dressing, the hypothesized microalgae-hydrogel patch could be applied to diabetic chronic wounds as it showed increased wound oxygenation, and angiogenesis, making it a novel medical innovation for the diabetic foot ulcers (Figure 3) (29, 31).

## Microalgae as a Source of Prebiotics Bioactive Molecules

Microalgae is an important source of prebiotics that defined as potential native and modified forms of polysaccharides (PS) such as xylooligosaccharides (XOS), galacto-oligosaccharides (GOS), alginate oligosaccharide (ALGOS), neoagaro-oligosaccharides (NAOS), galactans, arabinoxylans, and β-glucans (Table 3). These microalgae PS are against the digestion process by the metabolic enzymes in the human gut. Therefore, they could be used as dietary prebiotics and able to augment the growth of probiotics bacterial in the gut and colon (32, 33). de Medeiros et al. (34) used the prebiotic score (P. Score) technique to investigate the different tolerance abilities of microalgae against the digested through a method of an *in vitro* colonic fermentation. In that study, they mentioned that *A platensis* P. Score was 6.93 ± 0.05 and *C. vulgaris* was 2.54 ± 0.02 which were significantly high compared to the control with -1.35 ± 0.04.

**TABLE 3:** The different percentages of carbohydrate (CH), starch (ST), dietary fiber (DF), xylooligosaccharides (XOS), and galactooligosaccharides (GOS) originated from different microalgae species.

Microalgae species	CH (%)	ST (%)	DF (%)	XOS (%)	GOS (%)	References
*Chlorella vulgaris*	55.0	37.0	1.4	7–19	14–26	(35–37)
*Chlamydomonas reinhardtii*	60.0	55.0	–	–	4.5	(35)
*Chlorococcum sp.*	32.5	26.0	–	27.0	9.0	(35)
*Scenedesmus acutiformis*	36.9	16.4	–	–	–	(25, 35)
*Scenedesmus obliquus*	51.8	24.0	–	7.0	13.0	(35, 38, 39)
*Tetraselmis sp.*	26.0	28.0	–	–	–	(35, 40)
*Spirulina platensis*	54.4	2.7	0.4	5.4	13.3	(35, 41, 42)
*Nitzchia Closterium*	32.6	–	–	7.0	18.4	(35)
*Phaeodactylum tricornutum*	21.0	79.8	45.6	7.5	8.9	(35, 43, 44)
*Dunaliella tertiolecta*	85.3	70.0	–	1.0	1.1	(35, 45)
*Galdieria sulphuraria*	21.7	–	0.5–0.6	–	–	(46, 47)

Prebiotic was described by the Association of Probiotics and Prebiotics (ISAPP) as a non-digestible carbohydrate group, such as short and long-chain β-fructans [Fructooligosaccharide (FOS) and inulin], and GOS that beneficially affects the human by stimulating the growth of probiotics bacteria (like Lactobacillus spp., Bifidobacterium spp., Streptococcus spp., Lactococcus spp., and Saccharomyces spp.), and release some essential metabolites that could improve the human gut health (e.g., enhance the gut permeability through the released butyric acid) (48). These beneficial compounds are classified based on their resistance to the acidic pH and hydrolyze enzymes, absorption in the gastrointestinal tract, and fermentation potential by the intestinal microbiota (49). For instance, oligosaccharide carbohydrates (OSCs) are a good prebiotic family-like Fructans that consists of inulin and FOS or oligofructose. Their structure is a linear chain of fructose with β(2→1) linkage (50). In which, the specific structure of these molecules like, the length of fructans chain, is play a key role in their bacterial selection that can ferment them to the beneficial secondary metabolites (51).

**FIGURE 4:**
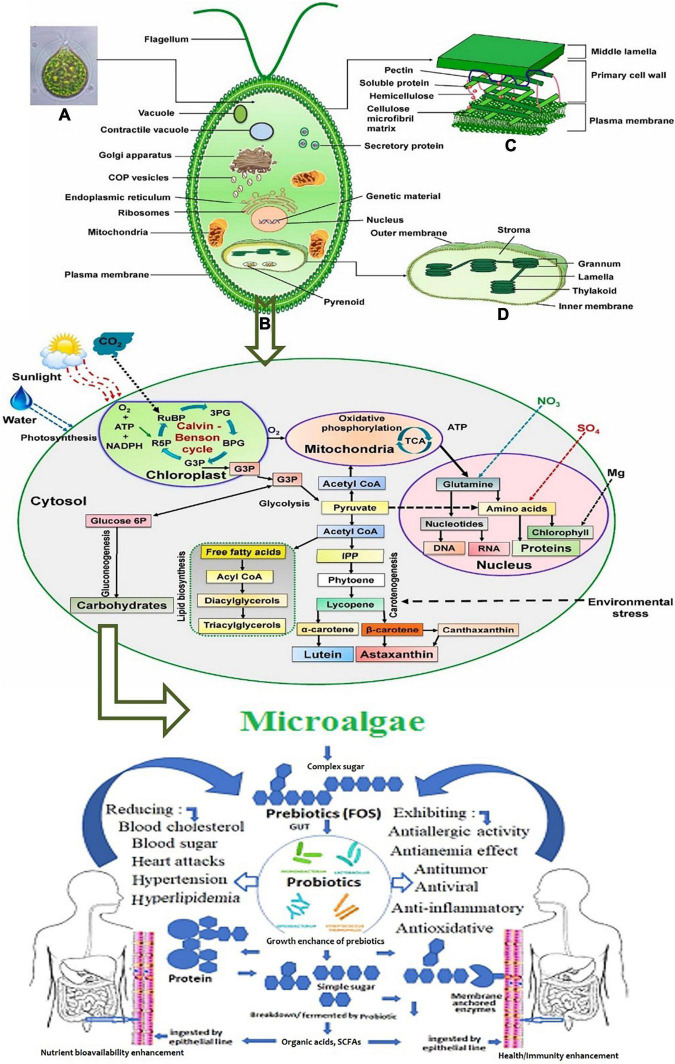
Systematic representation of metabolic pathways in unicellular microalgae for the synthesis of prebiotics. **(A)** The metabolic process of biomolecules production. **(B)** The benefits of the produced prebiotics as functional compounds for human health (52). Figure 1 schematic illustration of eukaryotic microalgae: **(A)** microscopic image, **(B)** cellular component and organelles **(C)** cell wall structure, and **(D)** chloroplast structure. Adopted and modified from Mehariya, Goswami, Karthikeysan and Verma (52) (Copyright permission: 5179190025925).

Figure 4 presents the systematic metabolic pathways in unicellular microalgae for the synthesis of prebiotics and their potential benefits as functional prebiotics for human health (52). In microalgae, PS synthesis occurs in the Golgi apparatus whereas it is cytoplasmic for Cyanobacteria (53). The main stages of EPS synthesis in microalgae and cyanobacteria are the production of activated monomer sugars, then their assembly by enzymes like glycosyltransferases that can produce polymers from these monomers in the extracellular compartment, as the meaning of EPS expression (54).

### Microalgae Functional Polysaccharides and Exopolysaccharides

**FIGURE 5:**
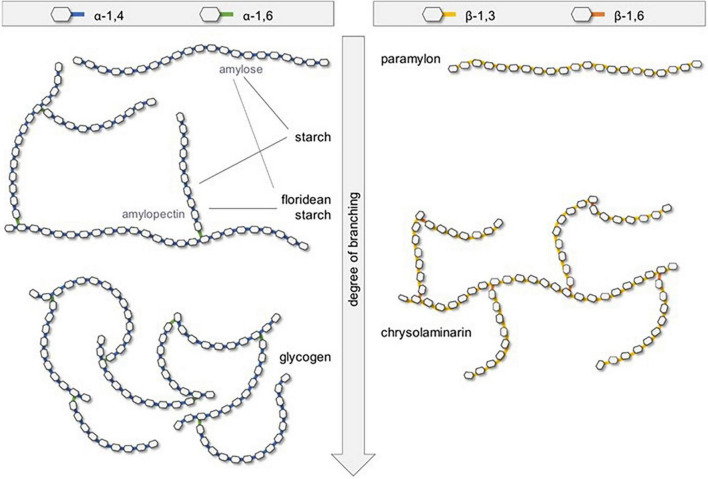
Schematic representation of the five common storage types of microalgae polysaccharides (121) (Copyright permission: 5179150775488).

Figure 5 schematic representation of the five common storage types of microalgae polysaccharides, in which the polysaccharides from microalgae can be distinguished into three main groups: intracellular, structural, and extracellular polymeric substances or exopolysaccharides (EPS) like extracellular glycans that serve for the cell-protective roles. In which, a few reports regarding the use of microalgae non-digestible EPSs as prebiotics. Hongpattarakere et al. (55) mentioned that EPS are long-chain polysaccharides that are secreted by microalgae into their surrounding during growth to protect them against desiccation. For instance, cell-bound polysaccharides (BPS) can be subdivided into different substructures that can form the colloidal EPS (53). These molecules exhibit high resistance to human gastrointestinal digestion, thus they enhance the activity of the beneficial bacteria colonized in the colon in the same way to other prebiotics (55). Schaper et al. (56) reported that EPS from Chlorophyta are mainly composed of Gal (Chlorella) or Glc (Chlamydomonas). And, cyanobacteria mainly produce Glc-rich EPS (57, 58). Combining ecological, biochemical, genomic, and transcriptomic approaches should provide significant advances in the understanding of EPS microalgae production, for both industrial uses and evolution comprehension (56).

Gurpilhares et al. (59) mentioned that *A. platensis* extracellular products (prebiotic, like EPS) are greatly contributing to the growth of the human gut microbiota. When these compounds are ingested they reach the colon without any change and then the microbial consortia of the human gastrointestinal promote their degradation. Shankar et al. (60) reported that the bacterial fermentation process with the non-digestible prebiotics in the human gut produces short-chain fatty acids (SCFA) like acetate, propionate, butyrate, and lactate. These SCFAs are reducing the pH level in the stomach and in the small intestine that is playing a vital role in inhibiting the growth of Gram-negative pathogens through the dissociation of the acids and production of anions in the bacterial cells. Also, these compounds are playing a vital role in colon health. For instance, butyric acid or butyrate is acting as a key energy source for the colonic epithelium and is acting as a protective agent against tumorigenesis of the epithelial cells (60). Moreover, there is a kind of starch produced by the microalgae called resistant starch (RS) and algaenan polymers that present in the microalgae cell wall and that are very resistant to the enzymatic hydrolysis in the human’s body (61). These resistant polymers can produce a high level of butyrate. Therefore, they could be considered as novel prebiotic fibers.

### Microalgae Functional Oligosaccharides

Some oligosaccharides are originated from a polysaccharide known as pectin and which could be considered as prebiotic polysaccharides. This type of oligosaccharide is well known as pectic oligosaccharide (POS). These compounds have the extension of homogalacturonan or rhamnogalacturonan I that could be acetylated at C2 or C3 that facilitate the linking with arabinose, galactose, and xylose sugars on the side chains and forming new structures of POSs. Also, GOS (produced from lactose sugar extension) is formed from galactose extension at C3, C4, or C6 from the originated sugar from lactose through enzymatic trans-glycosylation that form pentasaccharides with galactose in β(1→6), β(1→3), and β(1→4) to form different types of GOS such as trans-galactooligosaccharides (TOS) (49). Table 3 shows the different percentages of GOS and xylooligosaccharides (XOS) originating from different microalgae species. Valcheva and Dieleman (62) reported that 18 g of α-GOS for fourteen days were decreased the plasma lipopolysaccharide (LPS) concentrations of overweight human adults. Additionally, GOSs are stimulating several probiotic bacteria like Bifidobacteria and Lactobacilli. Also, Enterobacteria and Bacteroidetes are stimulated by GOS, but significantly lower than Bifidobacteria.

## Effective Extraction Methods for Microalgae Functional Prebiotics

**FIGURE 6:**
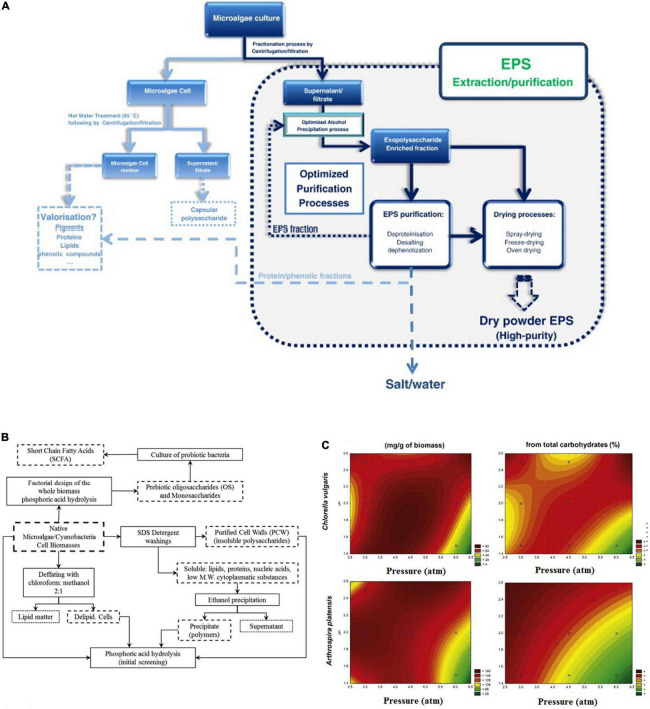
Extraction and purification of bioactive prebiotics. **(A)** Traditional strategy for the extraction and purification of exopolysaccharides from microalgae. **(B)** Thermophosphoric hydrolysis preparation strategy for the extraction and purification of microalgae oligosaccharides. **(C)** Contour plots for oligosaccharide production and purity via thermophosphoric hydrolysis of two microalgae species (53, 65) (Copyright permissions: 5179210453569, 5179200237659).

In this section, we will discuss in brief the recent commonly used methods that could be applicable for microalgae prebiotic extraction. These techniques have benefits and limitations. Figure 6A shows the method and common protocol for the extraction and purification of exopolysaccharides from microalgae.

### Chemical Dependent Extraction Methods

The chemical hydrolysis/pre-treatment followed by the alkaline extraction method is a possible methodology to obtain alginates the major polysaccharide in microalgae. The acid pre-treatment promotes the solubilization of the alginate and by alkaline extraction, soluble sodium alginate is formed and could be easily separated by the centrifugation process. However, this method is considered a time-consuming and complicated technique (63). Another chemical extraction method used solvents shown to be selective and more efficient. On the other hand, these methods showed toxicity coming from the used solvents like hexane, diethyl ether, benzene, and acetonitrile which are not eco-friendly chemicals (64). Leal et al. (65) fabricated a method for extracting the cell wall polysaccharides and oligosaccharides of the microalgae biomass by using were partially hydrolyzed with phosphoric acid (Figure 6B). They mentioned that the hydrolysis phosphoric acid catalyzes does not require any chemical removal, but just this weak acid simple neutralization. Besides, that acid-catalyzed hydrothermal pretreatment of microalgae obtained high concentrations of the extracted oligosaccharides (Figure 6C). Therefore, these methods which use weak acids like phosphoric or citric could be considered as eco-friendly chemical methods for microalgae polysaccharides and oligosaccharides. Because, they are not using hazardous solvents, and also generate small amounts of byproducts compared to the strong acids (65, 66).

### Green Extraction Methods

The green extraction methods brought the attention of the scientific world because of their safety as physical methods and alternatives to chemical methods. For instance, ultrasound (US)-assisted extraction, which is based on sound wave migration that generates cavitations, leads to the disruption of cells and their walls (67). Compared to other technologies, US technologies show high potential in the field of herbal science due to several unique advantages found in the US. From a financial point of view, it is easier and less expensive to scale up the US technology compared to other techniques like a microwave (MW), pulsed electric fields (PEF), high voltage electric field (HVEF), and high-pressure processing (HPP) methods (68). Further, the technology generates a better yield and thus improves the economics of the extraction process. On the other hand, combined emerging technologies (e.g., US and HPP) are considered superior to individual methods alone. The application of US not only enhances the extraction efficiency of the microalgae polysaccharides but also it can enhance the functional properties of these extracted macromolecules. For instance, Zhao et al. (69) showed that the United States has a significant effect on polysaccharides extracted from plant cells, and at the optimum condition it should have higher bioactivity like antioxidant activity. Joshi and Gogate (70) reported that US horn (20 kHz, 100 W, and 60°C) enhanced the acid hydrolysis for the production of reducing sugars. In which, it reduced the reaction time from 120 to 60 min with a high yield of reducing sugars (24.75 g/L). Also, they reported that US combined with oxidants like H_2_O_2_ could effectively decrease the hydrolysis time by acid that is used for targeting the plant polysaccharides by facilitating the lignin fraction, which increases the rate of reducing sugars production from plants (70).

Enzyme-assisted extraction has emerged as a promising tool to obtain extracts with attractive biological properties. The hydrolysis enzymes are effectively acting on the microalgae cell walls which facilitates the release of the intracellular prebiotics. Additionally, the enzymatic treatments have been used for the modification of obtained natural PS polymers that are used for the chemical structural evaluation of the novel prebiotic fibers and the enhancement of their biological activity, simplifying structural, and structure/function studies. The huge benefits of the enzymatic methods for the extraction of prebiotics are due to the decrease in the extraction time, the consumed energy, the used solvents, and the increase in the extracted yield (59). These advantages could be directed to its application for microalgae polysaccharides and oligosaccharides extraction and modification. Some types of microbial enzymes have been used for hydrolysis of microalgae polysaccharides, but they are mainly applied in lignocellulosic biomass conversion to fermented sugars. The difference between PS and oligosaccharides is due to the degree of polymerization (DP). In general, PS are molecules with a DP higher than 20 –25, while OS contain 2 –10 residues of sugars. Therefore, the oligosaccharides could be produced by using specific enzyme treatments on the polysaccharide chains. For example, alginate is the main PS of microalgae, and that contains polymannuronic acid, polyguluronic acid, and a mixture of polymannuronic acid and polyguluronic acid with a linear structure (also referred to as alginic acid) (53, 71). Zhu et al. (72) reported that using the enzymatic hydrolysis for the salt form of microalgae alginate (sodium alginate) to produce alginate oligosaccharides are increasingly used due to the formed OS excellent physicochemical properties.

## Methods for Identification of Microalgae Prebiotics Macromolecules

Microalgae macromolecule analysis has been practiced for decades. A promising strategy for microalgae chemical assessment is using bioinformatics to provide a fast prediction tool for its carbohydrates, proteins, and even lipids. Nowadays, a potential limitation is the lack of reference structures of some microalgae proteins, and thus a wide range of analytical methods should be used for building a strong database based on these different analytical destructive or non-destructive techniques combined with chemometrics and other informatics methods.

### Chemical-Free Non-destructive Methods

Spectroscopic methods for determining the chemical composition of microalgae have grown in popularity. Raman micro-spectroscopy, for example, is a quick, chemical-free, and non-invasive tool for characterizing single-cell molecules and their activities by detecting the studied molecules groups’ vibrational frequency in response to the laser beam exposer at specific wavenumbers like 350–600 nm for microalgae skeletal pyranose ring, glycosidic stretching CH_2_ and C-OH deformations of its polysaccharides (2, 15, 73).

#### Spectroscopic Methods for Microalgae Physicochemical Structure

##### Raman Micro-Spectroscopy of Single-Cell Microalgae

Raman micro-spectroscopy is a quick, label-free, and non-destructive technology that is used for the characterization of single-cell chemical construction and their bioactivities through information frequency vibration of the functional molecules by laser light inelastic scattering (15). Also, Raman spectroscopy has been used in many aspects of single microalgae research, such as chemical imaging of microalgae biochemical molecules like studying of their nutritional status (74), chemical imaging of its biochemical contents like carotenoids (75) and astaxanthin (76), and its antioxidant functionality (2). On the other hand, Raman responses are interfering with the fluorescence scattering chromophores coming from microalgae dyes like chlorophyll green pigment (77). Thus, deep learning and machine learning algorisms are frequently used for chemometric analyses and to obtain more-reliable information through building models with the standard chemical methods (78).

**FIGURE 7:**
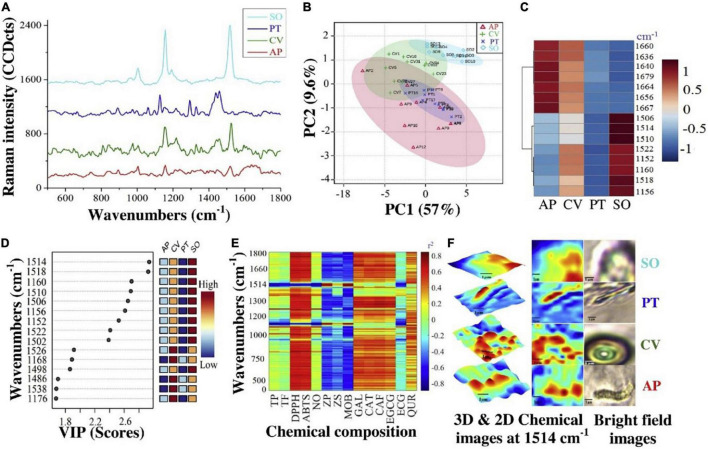
Single-cell Raman micro spectrometry chemical composition of *A. platensis* (AP), *C. vulgaris* (CV), *P. tricornutum* (PT), and *S. obliquus* (SO). **(A)** Full spectra, **(B)** PCA, **(C)** clustering heatmap, **(D)** VIP, **(E)** correlation (r^2^) summarization, and **(F)** 3D & 2D chemical images at 1514 cm^–1^ (2) (Copyright permission: 5179160871508).

Figure 7 shows a practical example of using micro-Raman in the field of single-cell microalgae detection for tracking of its macro and micromolecules. In that study, *A. platensis* (AP), *C. vulgaris* (CV), *P. tricornutum* (PT), and *S. obliquus* (SO) were fully scanned (400–1800 cm^–1^) by confocal Raman microspectroscopy. In which, by principal component (PC)1,2 and clustering heatmap, it was found that CV and SO were clustered as the same group compared to AP and PT for the wavenumber 1160 cm^–1^ (Figures 7B,C). Li et al. (15), and Wei et al. (79) mentioned that 1052 cm^–1^ are matching to Polysaccharides C-C stretches which is mainly present in the polar phase of microalgae. By VIP top scores, 1160 cm^–1^ was among the top significant wavenumbers, in which, SO was the highest, and PT was the lowest (Figure 7D). Besides, Huang et al. (75) mentioned the positive relationship between microalgae micromolcules and the non-polar polymers measured by Raman spectroscopy.

##### Surface-Enhanced Raman Scattering Technique

The high sensitivity surface-enhanced Raman spectroscopy technique that uses nanoparticles like nano-gold or silver as sensors for enhancing the sensitivity of the Raman scattering at very low levels leads to a huge scientific revolution in this field (80). The technique has attracted considerable interest in the bio-detection of microalgae organic molecules (79, 81) due to its non-destructive and ultrasensitive features (82). This technique provides a “molecular fingerprint” that can be used to identify a molecule or verify its presence in a sample using its intrinsic signals. The technique has been found to have significant advantages, such as high affinity for molecules, high sensitivity, and fingerprint resolution, which can effectively enhance the signal strength up to 10 orders of magnitude (83). Due to these excellent features, Ramya et al. (84) demonstrated the use of SERS with silver Ag nanocolloids on microalgae growth. They successfully visualized the distribution of polysaccharides of the microalgae cells. In this application, glass-coated slides with nanoparticles such as Ag were used due to their wide utilization and their good sensitivity and absorption capacities.

**FIGURE 8:**
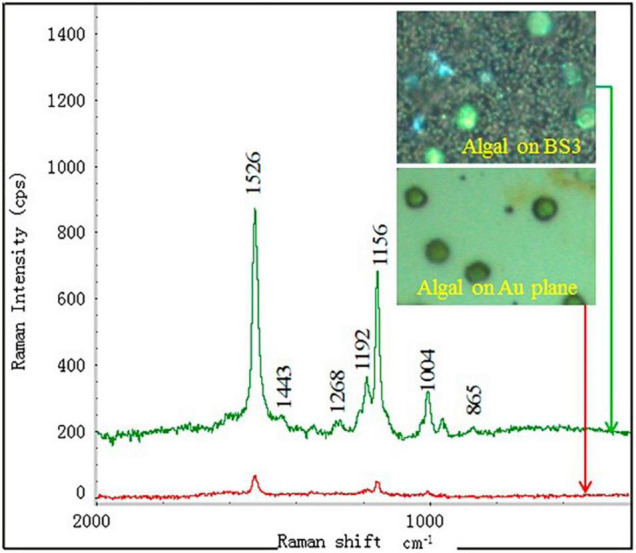
SERS detection of a single algal cell by using the BS3 substrate deposited with 400 nm gold layer. The inset are the images of the algal cells dispersed at the substrates taken from the optical microscope (86) (Copyright permission: 5179191297869).

Furthermore, Behrendt et al. (85) developed a “PhenoChip” which enhances the phenotyping of cyanobacteria and microalgae under the basis of multiparametric photo-physiological characterization and selection of unicellular phenotypes to be controlled by the user. Deng and Juang (86) developed a method based on nano-gold particles for the detection of microalgae chemical construction by SERS. They mentioned that SERS can detect a very low concentration of the chemical molecules at a concentration level as low as 100 ng/mL (Figure 8).

##### Microalgae Analysis by Fourier Transform Infrared Spectroscopy

Fourier transform infrared is a molecular spectroscopic technology that can identify the chemical groups based on their different absorbance of wavenumbers from 400–4000 cm^–1^. The cell macromolecule composition, (carbohydrate, lipid, and protein) have different characteristic ranges at different frequency regions of the FTIR spectrum. Therefore, it has the potential to be used for microalgal biochemical monitoring (87). Generally, FTIR spectroscopy is a non-destructive technique for polysaccharides and EPS structural characterization (88). The FTIR spectra are interpreted as vibrations of repeat structural. This method is commonly used for the assessment of conformational changes and structural dynamics of the microalgae polysaccharides (89). For instance, it has been shown that increasing the temperature affects the shape of the FTIR amide I band, which indicates the chemical conformation (90). Therefore, FTIR is regarded as one of the best non-destructive techniques for confirming protein structural features of aggregated or insoluble proteins. Rashmi et al. (90) mentioned that microalgae EPS of *S. elongatus* is attributed at 1400–1637 cm^–1^ for C = O shift, 3464 cm^–1^ for N–H stretch and 3779–3925 cm^–1^ for O–H stretch. In which, they reported that microalgae EPS are composed of at least one uronic acid with several sugar molecules. In that study, EPS of *S. elongatus* was found to possess 22% sugars and 8% uronic acid as a high molecular weight polysaccharide. Additionally, Goo et al. (89) used FTIR for characterization of *Dunaliella tertiolecta* EPS and comparing its unique structure with the potato amylose. They mentioned that 950–1200 cm^–1^ could be assigned as the best spectra regions of microalgae carbohydrates C-O and C-C stretching vibrations. By contrast, less pronounced IR bands at 890–900 cm^–1^ and 822 cm^–1^ were found only for EPS that related to β-d-glucans and (1 → 3)-α-d-glucans. Also, they mentioned that IR bands in the region of 800–950 cm^–1^ are very sensitive to anomeric configuration of glucose. The changes at 860–862 cm^–1^ indicates the changes in the α-configuration of glucose units. Thus in that study they distinguished the microalgae EPS structure from amylose close structure.

#### Electrochemical Gold Nanosensor Microelectrodes in Microalgae Single Cell Studies

**FIGURE 9:**
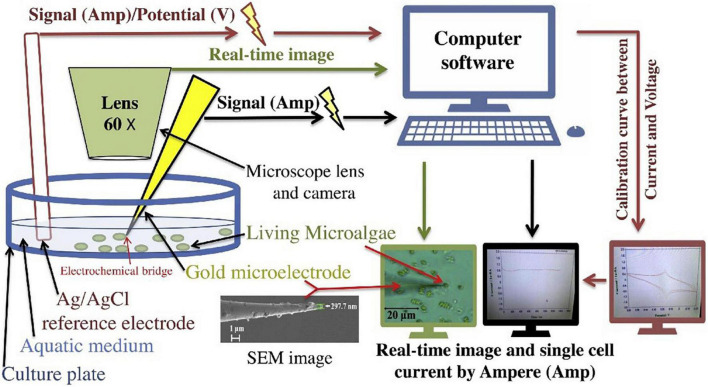
Illustration of microalgae single-cell electric current analytical detection by gold microelectrode (2) (Copyright permission: 5179160871508).

Single-cell electrochemical current by microelectrode has developed as an important technique for major studies of single-cell functionality (91). It is known that the combination of biomolecules with nanoparticles like gold nanoparticles creates interesting features for the development of nanosensors (92). Gouda et al. (2) fabricated a new method for detecting microalgae single-cell antioxidants through its single-cell electrochemical current through using a gold nanoprobe and three microelectrodes single-cell system (Figure 9). The most commonly used metal nanoparticles for fabricating these kinds of microelectrodes are nano-gold or silver for their high detection sensitivity (93). In which, according to Arias et al. (94), the uronic acids and sulfate contribute to the anionic nature of the microalgae EPS, conferring a negative charge outside the microalgae single cell. The critical dimension of these kinds of microelectrodes is so small with nano-diameters that it could be used to detect cellular biomolecule activities (95). Also, gold nanoprobe biosensors can be used in micro space electrochemistry and its pulled end can be precisely controlled down to 50 nm of attachment with the single-cell (96, 97). Recently, nanometer-sized electrodes were used to record intracellular ROS that are released by the metabolic pathways of the microalgae macromolecules which release some prebiotics like the released EPS from these cellular processes (92, 98, 99).

However, specific care is needed to maintain a very small current response (10^–10^ Amp) relation with the actual biochemistry of cells. Therefore, the continuous development of intracellular electrochemical detection and its relationship with chemical invasive methods and other non-invasive methods like micro-Raman spectroscopy should be more solidified.

#### Acoustic Sensors and Biosensors

**FIGURE 10:**
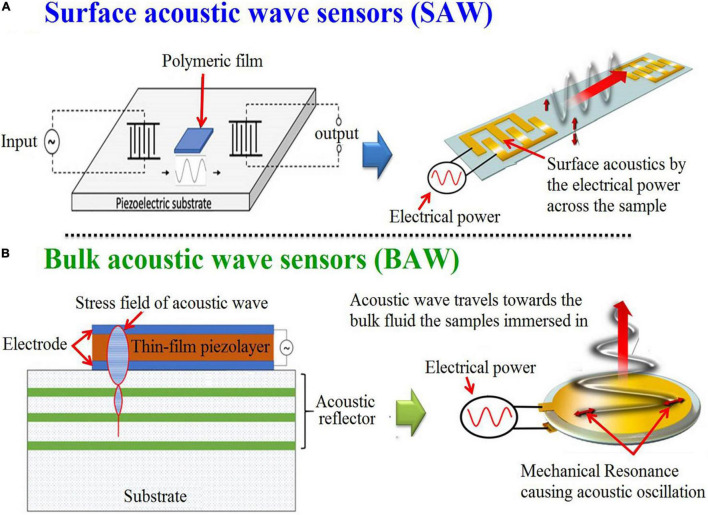
Graphic depicting in general terms the processes for the generation of surface **(A)** and bulk **(B)** acoustic wave sensors (102) (Open access and no requirement of Copyright permission).

The use of the United States for analyzing the microalgae composition and drawing chemical images of their structure, and visualizing their biomolecules has emerged as a hot scientific research topic. An acoustic wave sensor typically consists of a piezoelectric substrate (like quartz), covered with sensing polymeric film, and two interdigital transducers for input and output are commonly used for acoustic sensors chemical composition devices (100). In which, there are different types of these sensors [e.g., Micro/nano-acoustic biosensors, Surface acoustic wave sensors (SAW), and Bulk acoustic wave sensors (BAW)] (101, 102). The acoustic waves that are moving on the surface of the substrate are called SAW, while the waves that go through the substrate are called BAW (Figure 10) (103).

**FIGURE 11:**
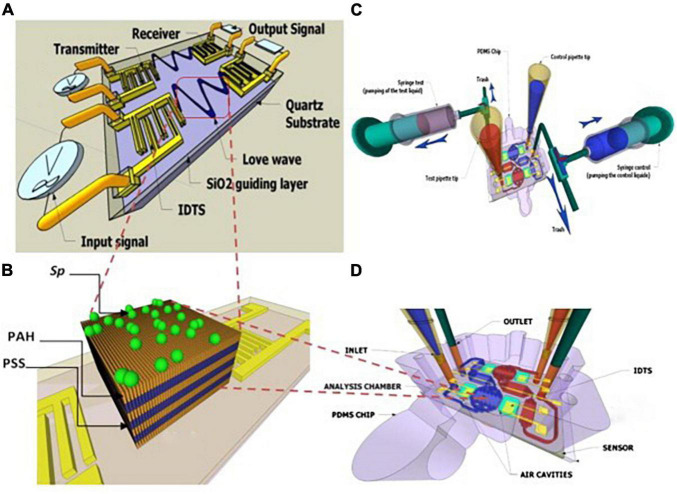
Schematic of a SAW sensor with a hybrid biofilm of polyelectrolyte microalgae. **(A)** Scheme of SAW. **(B)** *Spirulina* immobilization on a polyelectrolyte multilayer (PEM) coated with a layer by layer (LBL) method. **(C,D)** hydrodynamic chip with microfluidic network, aligned on SAW (104) (Copyright permission: 5179190742544).

For example, Tekaya et al. (104) used SAW for real-time tracking of *Arthrospira plantensis* microalgae biofilm on microfluidic chip. They mentioned that SAW allowed the optimization of the deposition process of microalgae biofilm for sensitive detection of microalgae elements. In that study, they characterized the toxicity of *A. plantensis* microalgae heavy metal (Cd^2+^ and Hg^2+^) with a detection limit 10^–12^ M (Figure 11). On the other hand, there are still some limitations to these kinds of sensors. For example, the complex circuitry, poor signal-to-noise ratio, and the humidity influences (100).

Nano-acoustic biosensors have been established to enhance the bioactivity of specific biomolecules, like enzymes, to increase detection sensitivity. These biosensors use air-filled protein nanostructures called gas vesicles that vibrate in response to ultrasound vibrational waves (105). The principle of using acoustic-based biosensors is based on linking the measurement criteria (like adsorption) as a modulation of the physical characteristics of ultrasound waves (like United States frequency and velocity), which is correlated with the concentration of analyte in the samples (101). Gouda (102) mentioned that solvothermal reaction was used for bio-imaging of plants by using quantum dots technology. The authors suggested that the viability of the technique could be used for *in vitro* cell imaging and *in vivo* imaging of natural plants.

### Chemical Dependent Destructive Methods

The analysis of microalgae macromolecules such as proteins, carbohydrates, and lipids using mass spectrometry is a very promising technique for multi-detection and in-depth characterization of these molecules’ physicochemical properties (8, 106). Also, chromatography-dependent methods enable researchers to separate these important molecules, identify their properties, and determine their amounts (107).

#### Mass Spectrometry Methods

The analysis of microalgae macro and micro hydrocarbon molecules using mass spectrometry can determine the primary sequence, post-processing modifications, molecular interactions, and structural studies of molecules like proteins. In which it may help in identifying and modifying their composition due to treatments (e.g., thermal and non-thermal treatments, and processing storage under modified environment. and so on). Mass spectrometers are composed of an ion source, a mass analyzer, and a detector, and various designs and modes of action are available for different applications. Electrospray ionization (ESI) technique (108) and matrix-assisted laser desorption ionization (MALDI) (109) are the most common mass spectrometers for microalgae biomass and prebiotic fibers characterization. The MALDI technology observes singly charged ions, whereas the ESI generally induces multiply charged states. Surface-enhanced laser desorption ionization (SELDI) is a modified technique of the MALDI technology that introduces a further purification step on the probe surface before the MS analysis (110). There are five kinds of mass analyzers commonly used in studying biofuel macromolecules. These include time-of-flight (TOF) mass, ion traps (IT) mass, quadrupoles (Q) mass, and Fourier transform ion cyclotron resonance (FTICR) mass (111–113). The mass analyzers can be combined in tandem MS (MS-MS) such as Q-TOF, Q-IT, TOF-TOF, and ITFTICR (114).

Advanced sequencing methods combine sequencing experiments of fractionated polysaccharides and oligosaccharides that are treated with hydrolyzed enzymes to generate smaller fractions and the database search could obtain the structure of the novel prebiotics obtained from microalgae (115). Also, the recent development of Multiple Reaction Monitoring mass spectrometry (MRM–MS) has made significant progress in the assessment and quantification of biofuel macromolecules. Specific peptide signatures can be quantified by MRM and the use of internal labeled peptide standards (116). Relative and absolute quantitation was achieved by using multiple reaction monitoring (MRM) with isotope-labeled peptides as internal standards.

#### Chromatographic Approaches

**FIGURE 12:**
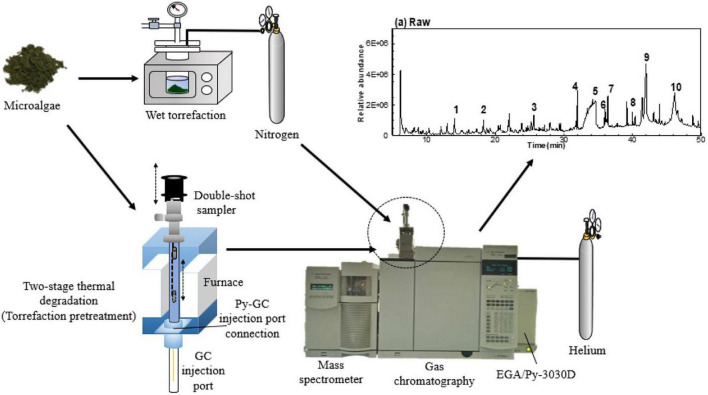
The experiment procedures of GC-MS analysis on wet microalgae and two-stage thermal degradation of microalgae (123) (Copyright permission: 5179191087529).

Chromatography enables researchers to separate components of a mixture, identifying their properties, and determining their amounts (5, 117, 118). Preparation of the proteins through the elimination of contaminants and separation of complex mixtures before mass spectrometer analysis is required to reduce the matrix complexity. Recently, there has been an increasing number of publications describing the use of LC-MS methods in the microalgae field for the characterization of their bioactive prebiotics (119). Also, the gas chromatography-mass spectrometry (GC–MS) technique is a powerful approach for the analysis of microalgae functional processes like pyrolysis. Figure 12 presents the setup of GC-MS of microalgae in single and double-shot pyrolysis. For instance, Rashmi et al. (90). The monosaccharide’s profile of sugar moiety of EPS of *S. elongatus* showed the presence of rhamnose, xylose, arabinose, fructose, sucrose, and maltose as identified by HPLC analysis. In the tested EPS of the *S. elongatus*, presence of rhamnose sugar indicates the component of rhamnolipid, one of the glycolipid may contribute to biosurfactant property. These metabolic bioactive phenomena is increasing the functionility of microalgae EPS as prebiotic sources.

Other chromatographic examples include ion-exchange chromatography, which allows separation based on the charge of molecules. Elution is carried out by changing the ionic strength of the mobile phase, either by modifying pH or increasing salt concentration. On the other hand, reverse phase chromatography is based on repulsive hydrophobic forces from the interactions between a polar carrier solvent that separate the microalgae macromolecules and the non-polar stationary phase into the column. Another chromatographic example is Anion Exchange Chromatography that could monitor the hydrolysis processes on the microalgae lignocellulose, and the target long-chain polysaccharides (120).

**FIGURE 13:**
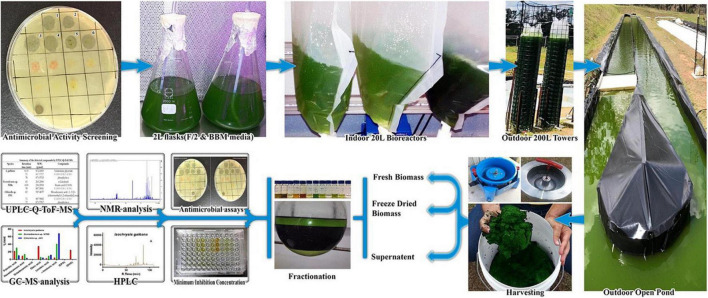
Using chromatographic and mass spectroscopy combined methods for the evaluation of microalgae antimicrobial compounds (20) (Copyright permission: 5179190549574).

Alsenani et al. (20) combined chromatographic and mass spectrometry with antimicrobial analyses for the evaluation of microalgae components’ antimicrobial activity to construct new bioactive phytochemical compounds for the food and pharmaceutical industries (Figure 13). In that study, the authors evaluated the antimicrobial activity of the microalgae species against pathogens using several extraction and measurement assays. By using a mixture of different chromatography techniques, like GC–MS and ultra-high-performance liquid chromatography-quadrupole time-of-flight mass spectrometry (UHPLC-Q-TOF-MS). They concluded that these methods had a high potential to separate bioactive compounds from microalgae species. These separated compounds were dominant and responsible for the inhibitory activity against pathogenic bacteria.

## Conclusion and Future Remarks

In conclusion, microalgae chemical composition plays an important role in its novelty applications as alternative nutritious prebiotic sources. The advancement of commonly used extraction methods and the analytical techniques for measuring microalgae species’ chemical composition could increase their applicability and potential uses in the field of bioactive prebiotic application and functional evaluations. Which, enhances their application in pharmaceuticals, functional food, and all related scientific fields. These advancements could include the discovery of new microalgae bioactive prebiotics, as well as the study of the real-time functional activity of microalgae exopolysaccharides, oligosaccharides during microalgal cultivation, as a critical approach to be developed for microalgae biotechnology of these functional compounds. Furthermore, nanoprobe microelectrodes with gold and other nanoparticles such as silver have become one of the most important approaches to verifying the functionality of various microalgae (for example, their antioxidant activity) as well as evaluating their physiological status. As a result, both conventional and spectroscopic methods could be used to validate the health benefits of microalgae health functional potential and their bioactive components benefits.

## Author Contributions

MG: conceptualization, studied the literature, drafted, and edited the manuscript. MT, YZ, and FF: helped in the writing process. XL and BC: helped in the writing process and the project administration. YH: supervision, writing, and reviewing. All authors edited, proofread, and accepted the final manuscript.

## Conflict of Interest

The authors declare that the research was conducted in the absence of any commercial or financial relationships that could be construed as a potential conflict of interest.

## Publisher’s Note

All claims expressed in this article are solely those of the authors and do not necessarily represent those of their affiliated organizations, or those of the publisher, the editors and the reviewers. Any product that may be evaluated in this article, or claim that may be made by its manufacturer, is not guaranteed or endorsed by the publisher.

## Abbreviations

*A*. *platensis*: *Arthrospira platensis*
ALGOS: alginate oligosaccharide
BAW: bulk acoustic wave sensors
*C. vulgaris*: *Chlorella vulgaris*
DHA: docosahexaenoic acid
EPA: eicosapentaenoic acid
EPS: exopolysaccharides
ESI: electrospray ionization
FOS: fructooligosaccharide
FTICR: fourier transform ion cyclotron resonance
FTIR: fourier transform infrared
GC–MS: gas chromatography-mass spectrometry
GOS: galacto-oligosaccharides
GRAS: generally recognized as safe
HBO: hyperbaric oxygen
HPP: high-pressure processing
HVEF: high voltage electric field
ISAPP: association of probiotics and prebiotics
IT: ion traps
LPS: lipopolysaccharide
MALDI: matrix-assisted laser desorption ionization
MRM–MS: multiple reaction monitoring mass spectrometry
MW: microwave
NAOS: neoagaro-oligosaccharides
OSCs: oligosaccharide carbohydrates
P. Score: prebiotic score
*P. tricornutum*: *Phaeodactylum tricornutum*
PEF: pulsed electric fields
POS: pectic oligosaccharide
PS: polysaccharides
Q: quadrupoles
RS: resistant starch
*S. elongates*: *Synechococcus elongates*
SAW: surface acoustic wave sensors
SCFA: short-chain fatty acids
SELDI: surface-enhanced laser desorption ionization
SERS: surface-enhanced raman scattering
TDO: topical dissolved oxygen
TGO: topical gaseous oxygen
TOF: time-of-flight
TOS: trans-galactooligosaccharides
UHPLC-Q-TOF-MS: ultra-high-performance liquid chromatography-quadrupole time-of-flight mass spectrometry
US: ultrasound
XOS: xylooligosaccharides.
